# Androgen Deprivation Therapy Differentially Impacts Bone and Muscle in the Short Term in Physically Active Men With Prostate Cancer

**DOI:** 10.1002/jbm4.10573

**Published:** 2021-11-15

**Authors:** Naim M. Maalouf, Avneesh Chhabra, Jason Zafereo, Ross Querry, Dwight A. Towler, Uma J. Thakur, Joseph Frankl, John R. Poindexter, Bayan Mogharrabi, May Xac, Orhan K. Öz, Craig D. Rubin

**Affiliations:** ^1^ Charles and Jane Pak Center for Mineral Metabolism and Clinical Research University of Texas Southwestern Medical Center Dallas TX USA; ^2^ Department of Internal Medicine University of Texas Southwestern Medical Center Dallas TX USA; ^3^ Department of Radiology University of Texas Southwestern Medical Center Dallas TX USA; ^4^ Department of Physical Therapy University of Texas Southwestern Medical Center Dallas TX USA; ^5^ Medical School University of Texas Southwestern Medical Center Dallas TX USA

**Keywords:** ANDROGEN DEPRIVATION, BONE, MALE OSTEOPOROSIS, MUSCLE, PROSTATE CANCER

## Abstract

Androgen deprivation therapy (ADT) is a cornerstone of advanced prostate cancer (PCa) therapy. Its use is associated with a loss of bone mineral density (BMD) and a greater risk of falls and osteoporotic fractures. In this prospective cohort study, we examined the impact of ADT on muscle and bone strength in men initiating ADT for PCa. Participants were evaluated at three time points: immediately before (week 0), and 6 and 24 weeks after ADT initiation. Study measures included fasting blood levels (for markers of muscle and bone metabolic activity), MRI and QCT imaging (for muscle fat content, and bone density and architecture), and validated clinical tests of muscle strength and gait. Sixteen men completed all study visits. At baseline and throughout the study, participants exercised a median of four times/week, but still experienced weight gain (+2.0 kg at week 24 versus week 0, *p* = 0.004). Biochemically, all men sustained dramatic early and persistent reductions in sex hormones post‐ADT, along with a progressive and significant increase in serum C‐telopeptide of type I collagen (CTX, +84% at week 24 versus week 0). There was a trend for rise in serum sclerostin (*p* = 0.09) and interleukin 6 (IL‐6) (*p* = 0.08), but no significant change in serum myostatin (*p* = 0.99). Volumetric BMD by QCT declined significantly at the femoral neck (−3.7% at week 24 versus week 0), particularly at the trabecular compartment. On MRI, there were no significant changes in thigh muscle fat fraction. On physical testing, men developed weaker grip strength, but experienced no worsening in lower extremity and lumbar spine muscle strength, or on functional tests of gait. In conclusion, in physically active men, ADT for 24 weeks results in a significant increase in bone resorption and reduction in BMD, but nonsignificant changes in thigh muscle quality (on imaging) or strength and gait (on functional testing). © 2021 The Authors. *JBMR Plus* published by Wiley Periodicals LLC on behalf of American Society for Bone and Mineral Research.

## Introduction

Although androgen deprivation therapy (ADT) is a cornerstone of advanced prostate cancer (PCa) therapy, men receiving ADT are at increased risk of bone loss and subsequent fractures.^(^ ^1^, ^2^ ^)^ Use of anti‐resorptive therapy (bisphosphonates and denosumab) can reduce ADT‐related decline in bone mineral density (BMD).^(^ ^3^, ^4^ ^)^ Nevertheless, the risk of weakness, falls, and hip fractures in ADT recipients remain high.^(^ ^5^, ^6^ ^)^

Patients receiving ADT experience significant reduction in circulating levels of both androgens and estrogens.^(^ ^7^ ^)^ Androgen receptors are expressed on myocytes, and ADT‐related fall and fracture risk may be in part due to early changes in muscle and body composition. A crosstalk between bone and muscle (independent of loading) is well described in the literature,^(^ ^8^ ^)^ and preclinical studies have shown that sex steroids act on both of these tissues through similar molecular and cellular mechanisms.^(^ ^9^ ^)^ These findings raise the possibility that increased risk of fall and fracture may represent the final common pathway in the intersection between bone and muscle loss with ADT. On the other hand, androgenic steroids can significantly increase muscle but not bone mass in animals and humans, arguing against a direct role of androgens in the crosstalk between muscles and bones. Furthermore, although exercise can mitigate ADT‐induced changes in body composition, trials investigating the impact of exercise on the risk of fracture are limited.^(^ ^10^, ^11^ ^)^ These observations highlight the need to better understand the relationship between skeletal muscle and bone in humans.

Some of the changes in body composition observed with aging (increase in fat mass:lean mass ratio, preferential accumulation of visceral fat, and fat infiltration within the skeletal muscle) are similar to those of patients undergoing ADT, although they occur on a different timescale and to a different magnitude.^(^ ^12^ ^)^ Myokines, cytokines, and other circulating factors (eg, insulin‐like growth factor 1 [IGF‐1], interleukin 6 [IL‐6], IL‐15, Wingless/Integrated (Wnt) 3a, Wnt4) have emerged as global modulators of metabolic frailty that arises with aging, sarcopenia, and disuse atrophy, by regulating musculoskeletal remodeling, inflammation, fuel metabolism, and resilience.^(^ ^13^, ^14^, ^15^ ^)^ Similar pathways may be operating in the setting of ADT initiation. In this study, we measured some of these circulating factors because understanding their relation to muscle and bone strength may guide the development and tracking of pharmacologic and exercise interventions that preserve muscle and bone strength in ADT‐treated patients.

The proposed crosstalk between bone and muscle led us to prospectively examine the temporal changes in bone and muscle mass and metabolism in men initiating ADT for PCa to the test the hypothesis that a decline in muscle strength precedes the decline in bone strength in this population. We used a combination of serologic, radiologic, and functional tests to assess the musculoskeletal impact of androgen deprivation.

## Subjects and Methods

### Protocol overview

Men with PCa were recruited from the University of Texas (UT) Southwestern Urology and Radiation Oncology Clinics and enrolled prior to ADT initiation. Each participant was evaluated at three time points: immediately prior to first ADT dose (week 0), and at 6 and 24 weeks following ADT initiation. Study measures at each time point included fasting venous blood (for sex hormones and biochemical markers of muscle and bone metabolic activity), MRI and CT imaging studies (assessing muscle composition, and bone volume and density) and validated physical tests of gait and muscle strength. Gleason score was abstracted from the participant's chart, whereas body mass index (BMI) was calculated from measurements of body weight and height obtained at each of the study visits. Participants were also queried about the number of sessions of ≥20 minutes of moderate‐to‐higher intensity exercise weekly to determine whether they met the American College of Sports Medicine physical activity recommendations for older adults.^(^ ^16^ ^)^

The study protocol was approved by the UT Southwestern Institutional Review Board (IRB) and registered on the ClinicalTrials.gov website (ClinicalTrials.gov Identifier: NCT03386812). All participants provided informed consent.

### Participants

Men with PCa planning to initiate ADT were offered participation in this study. Inclusion criteria were age 55 to 89 years and ability to participate in physical testing. Exclusion criteria included presence of bone metastasis, expected surgery, contraindication to MRI, severe functional impairment (inability to perform basic activities of daily living on a sustained basis), chronic kidney disease (CKD) stage 4 or worse, severe anemia (hemoglobin <9 g/dL), or prior or planned use of anti‐resorptive agents (bisphosphonate, denosumab).

### Blood studies

Analytes measured on fasting blood included sex steroids—total and bioavailable testosterone, and free estradiol—by chromatography/mass spectrometry (Quest Nichols Institute, San Juan Capistrano, CA, USA). ELISA kits were used for the measurement of serum intact parathyroid hormone (PTH) (BioAmerica, Irvine, CA, USA); cross‐linked C‐telopeptide of type I collagen (CTX) (Immunodiagnostic Systems, Gaithersburg, MD, USA); insulin‐like growth factor 1 (IGF‐1), sclerostin, myostatin, and interleukin 6 (IL‐6) (R&D Systems, Minneapolis, MN, USA).

### Imaging studies

MRI of pelvis (from L_2_ level to upper thighs) was performed on a 3‐T MR scanner (Ingenia; Philips, Best, Netherlands), using a torsoXL coil linked to a spine coil. MRI parameters measured in the proximal quadriceps, gluteal, and psoas muscles included quantitative fat fraction analysis on two‐dimensional (2D) Dixon quant sequence and anatomy was cross‐checked with isotropic three‐dimensional (3D) T2 Dixon sequence reconstructed axially at the level immediately below the lesser trochanter to ensure standardization. The pelvic muscles were evaluated at fixed points in the transverse plane at the level of the sacroiliac joint. Dixon quant relies on the difference in chemical shift of protons within water and lipids to calculate fat fraction [ratio of fat signal to total signal (fat signal + water signal)] within a given voxel. Image reconstruction and measurements were done using HOROS software (HOROS Project; https://horosproject.org/) using a free hand region of interest (ROI) incorporating the whole muscle with careful exclusion of the regional fatty tissue.

Pelvic CT images including lower lumbar spine and hips were obtained on a IQon Spectral CT scanner (Philips North America Corporation, Cambridge MA, USA). Images were exported for quantitative analysis using QCT Pro (Mindways Software, Austin, TX, USA) to measure volumetric bone density at the spine, and volumetric bone density at the femoral neck and total hip. Measurements were made for the overall bone compartment, and in trabecular and cortical compartments separately. Femoral neck cortical surface area and cross‐sectional moment of inertia were calculated with the qCT Pro Bone Investigational Toolkit.

### Physical testing

Strength testing included measurement of hand grip strength (bilaterally) and lower extremity testing on the nondominant side. Hand grip measurements included maximal sustained hand grip (for 5 seconds), with three trials alternating between left and right side, and 10‐second rest between tests. Measurements were obtained using a JTech Echo electronic hand grip dynamometer and a Tracker 5 analysis software (JTech Medical, Midvale, UT, USA). Lower extremity strength measurements included isokinetic testing of four pairs of antagonistic muscle actions: hip flexion/extension; hip abduction/adduction; knee flexion/extension; and lumbar flexion/extension. Testing protocol included concentric force production under two trials of varying speed and repetitions: Trial 1 consisted of slow speed × 5 maximal repetitions (testing for maximal force production), with 2‐minute rest period between repetitions. Trial 2 consisted of faster speed × 10 maximal repetitions (to evaluate muscular endurance stress). Variables measured included peak torque (ft‐lbskg), total work (Watts), and coefficient of variation (%). Measurements were obtained on a Biodex System 4 (Biodex Medical Systems, Shirley, NY, USA).

Gait testing was done on a 20‐foot Zeno instrumented walkway with Pkmas software (Protokinetics, Havertown, PA, USA). The protocol consisted of 80 feet (4 × 20 feet) of linear walking during two separate trials: self‐selected normal walking velocity, and fastest safe walking velocity. To ensure a more stable constant velocity during the trials, participants began 4 feet behind mat to allow for normal acceleration to achieve steady state velocity on the mat and were instructed to walk completely off the mat before slowing down. Measured parameters included gait velocity (cm/s), step length (bilaterally and ratio), cadence (steps/min).

### Self‐report questionnaires

The 29‐item Patient‐Reported Outcomes Measurement Information System (PROMIS‐29), version 2.0 was used to assess health‐related quality of life across eight domains because it has been shown to be valid and reliable in a community‐dwelling, geriatric population.^(^ ^17^ ^)^ PROMIS‐29 T‐scores of 50 are considered average for the general population for all domains except pain intensity, which was expressed as a raw score between 0 and 10. Higher scores indicate greater impairment for the domains of anxiety, depression, fatigue, pain interference, pain intensity, and sleep disturbance, whereas higher scores in the physical functioning and social role domains represent lesser impairment. The 16‐item Activities‐Specific Balance Confidence (ABC) scale was used to assess for fall risk. Scores on the ABC range from 0 to 100, with higher scores indicating increased balance confidence and reduced risk of falls. The ABC has been shown to have excellent reliability and validity in a community‐dwelling, geriatric population.^(^ ^18^, ^19^ ^)^ A score <67% has been shown to accurately identify those who have a history of falls 84% of the time.^(^ ^20^ ^)^ The ABC and PROMIS‐29 were administered at baseline, 6 weeks, and 24 weeks after enrollment.

### Statistical methods

For this cohort pilot study to characterize the acute and subacute changes in serum chemistry, muscle, and bone, a power analysis was not applicable; precision of results was evaluated with 95% confidence intervals (CIs). Statistical analysis was descriptive, with CI estimation for changes between visits. Continuous outcomes were explored with linear mixed‐effects models for comparison of means between the three visits, and evaluation of the trajectory over study duration. Contrasts from these models were used to construct pairwise comparisons, least squares means and 95% CIs. All statistical analyses were performed using SAS 9.4 (SAS Institute, Cary, NC, USA). Two‐sided *p* values of <0.05 were considered statistically significant.

## Results

### Study participants

A total of 18 participants consented to participate in this study. Two participants completed the baseline studies but did not return for the follow‐up visits. The remainder of the results described hereafter are for the 16 participants who completed all study visits. Most participants were white (81.3%), and mean age was 67 years (standard deviation [SD] = 7). Mean ± SD presenting PSA was 8.0 ± 14.2 ng/mL, and mean Gleason score was 6.9 ± 0.4 on prostate biopsy. These men reported participating in a self‐selected exercise regimen consisting of ≥20 minutes of moderate intensity exercise a median of 4 days/week at baseline. Despite continued participation in regular self‐selected physical activity at a similar frequency throughout the study period, body weight and BMI increased significantly over the study duration (Table 1).

**Table 1:** Longitudinal Changes in Anthropometric Characteristics and Participation in Physical Activity During the Study

Characteristic	Baseline	Week 6	Week 24	*p*
Body weight (kg)	97.5 (92.0–103.1)	97.8 (92.3–103.4)	99.5 (94.0–105.1)^a^	0.004
BMI (kg/m^2^)	29.8 (28.2–31.4)	29.9 (28.3–31.5)	30.4 (28.8–32.0)^a^	0.003
Exercise participation (number of days/week)	3.6 (2.4–4.7)	3.2 (2.0–4.3)	3.3 (2.1–4.4)	0.69

Data are shown as least squares mean (95% CI).

BMI = body mass index; CI = confidence interval.

^a^ *p* < 0.05 in for week 24 versus week 0 using repeated measures analyses.

### Biochemical parameters

**Fig. 1:**
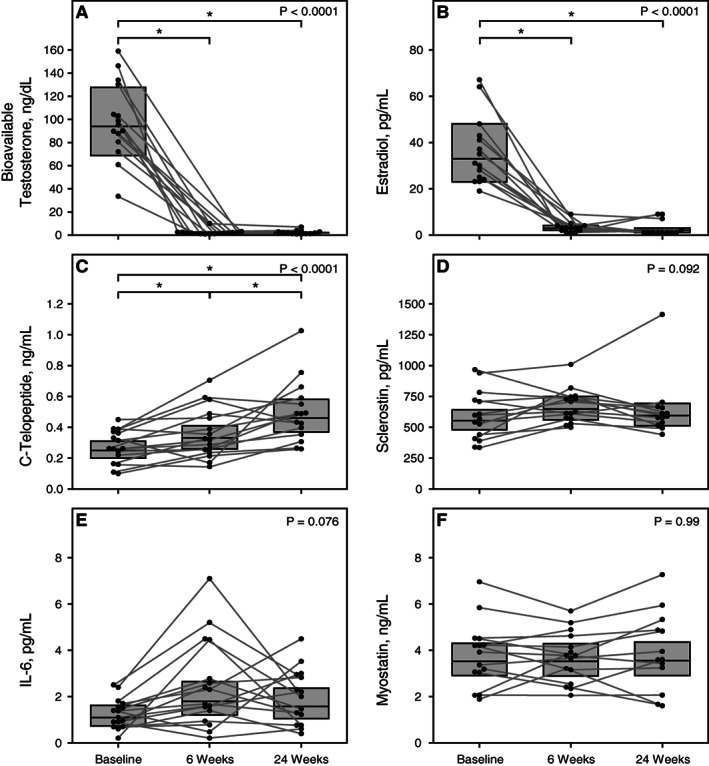
Changes in biochemical parameters during the study. (*A*) Serum bioavailable testosterone. (*B*) Serum estradiol. (*C*) CTX. (*D*) Serum sclerostin. (*E*) Serum IL‐6. (*F*). Serum myostatin. Data shown for individual study participants. Boxes indicate least square means with 95% CIs. Value of *p* at upper right corner represents *p* value from linear mixed‐effects models. *p < 0.05 using pairwise comparison. CI = confidence interval; CTX = serum C‐telopeptide; IL‐6 = interleukin 6.

Serum testosterone decreased markedly and significantly following ADT initiation (total testosterone: from 467 ng/dL at baseline to 9 ng/dL and 10 ng/dL at weeks 6 and 24, respectively; bioavailable testosterone: from 93.7 ng/dL at baseline to 1.6 ng/dL and 1.7 ng/dL at weeks 6 and 24, respectively (Fig. 1*A*); free testosterone: 47.0 pg/mL at baseline to 0.8 pg/mL and 0.9 pg/mL at weeks 6 and 24, respectively; *p* < 0.0001 for all) (Table 2). Consequently, circulating estradiol decreased from 33 pg/mL at baseline to 3 pg/mL and 2 pg/mL at weeks 6 and 24, respectively (Fig. 1*B*).

**Table 2:** Longitudinal Changes in Serum Analytes

Parameter	Baseline	Week 6	Week 24	*p*
Total testosterone (ng/dL)	467 (362–602)	9 (7–11)^b^	10 (8–13)^a^	<0.0001
Bioavailable testosterone (ng/dL)	93.7 (68.8–127.6)	1.6 (1.2–2.2)^b^	1.7 (1.3–2.4)^a^	<0.0001
Free testosterone (pg/mL)	47.0 (34.7–63.7)	0.8 (0.6–1.1)^b^	0.9 (0.6–1.2)^a^	<0.0001
Ultrasensitive estradiol (pg/mL)	33 (23–48)	3 (2–4)^b^	2 (1–3)^a^	<0.0001
C‐telopeptide (ng/mL)	0.25 (0.20–0.31)	0.33 (0.26–0.41)	0.46 (0.37–0.58)^a^	<0.0001
PTH (pg/mL)	55 (39–76)	35 (25–49)^b^	37 (27–52)^a^	0.023
IGF‐1 (ng/mL)	101 (88–116)	108 (94–124)	89 (77–103)	0.15
Myostatin (ng/mL)	3.53 (2.90–4.30)	3.52 (2.89–4.29)	3.56 (2.90–4.36)	0.99
IL‐6 (pg/mL)	1.09 (0.74–1.62)	1.80 (1.21–2.66)	1.58 (1.05–2.36)	0.076
Sclerostin (pg/mL)	555 (479–643)	648 (560–750)	595 (511–694)	0.092

Data are shown as least squares mean (95% CI).

CI = confidence interval; IGF‐1 = insulin‐like growth factor; IL‐6 = interleukin 6; PTH = parathyroid hormone.

^a^ *p* < 0.05 for week 24 versus week 0 using repeated measures analyses.

^b^ *p* < 0.05 for week 6 versus week 0 using repeated measures analyses.

Over the period of the study, serum CTX increased progressively and significantly (from 0.25 ng/mL at baseline to 0.33 ng/mL at 6 weeks and 0.46 ng/mL at 24 weeks) (Fig. 1*C*), whereas serum PTH decreased significantly (from 55 pg/mL at baseline to 35 pg/mL and 37 pg/mL at weeks 6 and 24, respectively). Although this did not reach statistical significance, there was a small rise in serum sclerostin (555 pg/mL at baseline to 648 pg/mL and 595 pg/mL at 6 and 24 weeks, respectively, *p* = 0.092, Fig. 1*D*) and serum IL‐6 (1.09 pg/mL at baseline to 1.80 g/mL and 1.58 pg/mL at 6 and 24 weeks respectively, *p* = 0.076, Fig. 1*E*). There were nonsignificant changes in serum myostatin (Fig. 1*F*) and IGF‐1 (Table 2).

### Imaging studies

Changes in BMD assessed by quantitative CT (QCT) scan are shown in Table 3. There was a significant decline in volumetric BMD (vBMD) at the femoral neck (from 272 mg/cm^3^ at baseline to 270 mg/cm^3^ at 6 weeks and 260 mg/cm^3^ at 24 weeks), corresponding to a 3.7% decline in femoral neck BMD at week 24. The decline in BMD was more prominent at the trabecular compartment (−6.1% at 24 weeks) than the cortical compartment of the femoral neck. Changes in vBMD at the spine (total bone compartment) and total hip (cortical, trabecular, and total bone component) were nonsignificant (Table 3). Cross‐sectional area and cross‐sectional moment of inertia of the femoral neck did not change significantly over the 24 weeks of the study.

**Table 3:** Longitudinal Changes in QCT and MRI‐Based Parameters

Parameter	Baseline	Week 6	Week 24	*p*
QCT				
Overall bone (trabecular + cortical)				
Femoral neck vBMD (mg/cm^3^)	272 (241–308)	270 (238–305)	260 (230–294)^a^	0.03
Total hip vBMD (mg/cm^3^)	279 (250–311)	276 (248–308)	271 (244–302)	0.21
Spine vBMD (mg/cm^3^)	106 (88–128)	106 (88–128)	102 (84–123)	0.16
Cortical bone compartment				
Femoral neck vBMD (mg/cm^3^)	1029 (938–1128)	1011 (922–1109)	1064 (970–1167)	0.12
Total hip vBMD (mg/cm^3^)	940 (906–976)	932 (898–967)	955 (920–991)	0.38
Trabecular bone compartment				
Femoral neck vBMD (mg/cm^3^)	132 (121–144)	129 (118–140)	124 (114–135)^a^	0.04
Total hip vBMD (mg/cm^3^)	131 (119–144)	130 (118–143)	126 (114–139)	0.09
MRI				
Muscle fat fraction (%)				
Gluteus	10.7 (9.2–12.5)	9.9 (8.5–11.5)	10.2 (8.8–11.8)	0.54
Iliopsoas	9.0 (7.0–11.8)	8.2 (6.3–10.6)	9.1 (7.0–11.9)	0.69
Quadriceps	7.5 (5.0–11.3)	8.8 (6.4–12.1)	9.4 (7.0–12.6)	0.50

Data are shown as least squares mean (95% CI).

CI = confidence interval; MRI = magnetic resonance imaging; QCT = quantitative computed tomography; vBMD = volumetric bone mineral density.

^a^ *p* < 0.05 for week 24 versus week 0 using repeated measures analyses.

MRI results showed no significant changes in the fat fraction in the gluteus, iliopsoas, and quadriceps muscles (Table 3).

### Strength testing

Participants experienced a significant decline in grip strength in their dominant hand, particularly in terms of peak force generated (Table 4). In contrast, there were nonsignificant changes in lower extremity strength (hip sagittal flexion, sagittal extension, and frontal abduction and adduction, and knee sagittal flexion and extension) tested in slow and fast mode. The only exception was a significant *increase* in sagittal hip flexion peak torque assessed under slow condition from 66 N m (95% CI, 53–82) at week 0 to 88 N m (95% CI, 72–109) at week 24. Lumbar spine muscle strength (trunk sagittal flexion and extension) remained unchanged (Table 4).

**Table 4:** Longitudinal Changes in Strength Testing Parameters

Parameter	Baseline	Week 6	Week 24	*p*
Grip strength peak force average (N m) (nondominant)	34.9 (31.6–38.5)	34.6 (31.4–38.2)	33.3 (30.2–36.7)	0.19
Grip strength peak force average (N m) (dominant)	38.4 (34.9–42.2)	37.7 (34.3–41.4)	35.4 (32.2–38.9)^a^	0.042
Sagittal hip flexion peak torque (N m) (fast)	80 (69–93)	85 (73–98)	87 (75–100)	0.53
Sagittal hip flexion peak torque (N m) (slow)	66 (53–82)	85 (69–105)^b^	88 (72–109)^a^	0.008
Sagittal hip extension peak torque (N m) (fast)	58 (44–75)	54 (42–70)	57 (44–74)	0.81
Sagittal hip extension peak torque (N m) (slow)	62 (45–85)	62 (45–85)	57 (42–78)	0.86
Sagittal knee flexion peak torque (N m) (fast)	43 (37–50)	40 (34–47)	40 (35–47)	0.27
Sagittal knee flexion peak torque (N m) (slow)	45 (36–55)	45 (36–56)	47 (38–58)	0.73
Sagittal knee extension peak torque (N m) (fast)	74 (66–83)	71 (63–80)	72 (64–81)	0.53
Sagittal knee extension peak torque (N m) (slow)	78 (59–102)	88 (67–116)	75 (57–99)	0.61
Frontal hip abduction peak torque (N m) (fast)	45 (35–59)	54 (42–69)	46 (36–59)	0.12
Frontal hip abduction peak torque (N m) (slow)	65 (49–87)	55 (42–74)	50 (37–67)	0.40
Frontal hip adduction peak torque (N m) (fast)	45 (37–54)	40 (33–48)	41 (34–50)	0.26
Frontal hip abduction peak torque (N m) (slow)	43 (36–51)	42 (36–50)	42 (35–50)	0.96
Sagittal trunk flexion peak torque (N m) (fast)	81 (69–96)	83 (70–99)	77 (65–91)	0.33
Sagittal trunk flexion peak torque (N m) (slow)	82 (66–101)	84 (68–104)	77 (62–95)	0.69
Sagittal trunk extension peak torque (N m) (fast)	119 (90–158)	132 (99–175)	115 (86–152)	0.44
Sagittal trunk extension peak torque (N m) (slow)	125 (94–165)	121 (92–161)	117 (88–154)	0.71

Data are shown as least squares mean (95% CI).

CI = confidence interval.

^a^ *p* < 0.05 for week 24 versus week 0 using repeated measures analyses.

^b^ *p* < 0.05 for week 6 versus week 0 using repeated measures analyses.

Over the 24 weeks of the study, there were nonsignificant changes in gait velocity and step length assessed both at self‐selected normal walking velocity and at fastest safe walking velocity (Table 5). There was a significant increase in cadence measured at fastest walking velocity (127 [95% CI, 121–134] steps/min at baseline to 136 [95% CI, 129–143] steps/min at week 24), but not in cadence at self‐selected speed.

**Table 5:** Longitudinal Changes in Gait Testing Parameters

Parameter	Baseline	Week 6	Week 24	*p*
Velocity, self‐selected speed (cm/s)	119 (109–130)	117 (107–128)	116 (107–127)	0.86
Velocity, fast speed (cm/s)	158 (145–172)	168 (154–182)	170 (156–185)	0.20
Step length, self‐selected speed (cm)	64 (59–69)	66 (61–71)	64 (60–69)	0.68
Step length, fast speed (cm)	74 (64–86)	84 (72–97)	74 (64–86)	0.35
Cadence, self‐selected speed (steps/min)	107 (79–146)	82 (60–111)	108 (79–147)	0.35
Cadence, fast speed (steps/min)	127 (121–134)	133 (126–140)	136 (129–143)^a^	0.049

Data are shown as least squares mean (95% CI).

CI = confidence interval.

^a^ *p* < 0.05 for week 24 versus baseline using repeated measures analyses.

### Self‐report questionnaires

At baseline, study participants’ scores were within the expected ranged for the general population for all domains tested by the PROMIS‐29 quality of life questionnaire (representing no significant abnormality in the anxiety, depression, fatigue, pain interference, pain intensity, sleep disturbance, physical functioning, and social role domains) (Supplemental Table S1). At weeks 6 and 24, these scores did not vary significantly from baseline. Participants’ median score in the ABC scale was 95.5 (on a scale of 0–100) at baseline, indicating high confidence in balance, and reduced risk of falls. The score did not vary significantly at the week 6 and week 24 time points (Supplemental Table S1).

## Discussion

ADT is well‐described to increase the risk of fall^(^ ^21^ ^)^ and fracture.^(^ ^22^ ^)^ In this prospective cohort study, we assessed early changes in bone and muscle strength in men initiating ADT for PCa treatment using a multidisciplinary approach. We found that ADT resulted in significant worsening in parameters reflecting bone health (elevation in serum CTX, decrease in vBMD at the femoral neck on QCT), but non‐significant changes in parameters reflecting muscle health (circulating myostatin, muscle fat content on MRI, lumbar and lower extremity muscle maximal strength performance, and typical straight line gait parameters). The regular regimen of physical activity that our study participants engaged in may have protected them from ADT‐related decline in maximal muscle strength but was insufficient to prevent against ADT‐induced accelerated bone resorption and loss of bone density.

ADT results in a significant increase in bone resorption and a reduction in BMD both in PCa‐treated men^(^ ^23^ ^)^ and healthy young men.^(^ ^24^ ^)^ We found a similar increase in the bone resorption marker serum CTX and decline in vBMD at the femoral neck. We observed a greater decline in trabecular compared to cortical vBMD with ADT similar to what was described in a cross‐sectional study comparing vBMD in ADT‐treated versus untreated men with PCa.^(^ ^25^ ^)^ This finding is supported by prospective studies showing a preferential improvement in trabecular BMD with testosterone supplementation in hypogonadal men.^(^ ^26^, ^27^ ^)^ This preferential loss in trabecular BMD is likely explained by the reduction in circulating estradiol with ADT, because estradiol levels have been previously significantly associated with trabecular but not cortical vBMD at the femoral neck.^(^ ^28^ ^)^ It is also possible that declines in cortical vBMD could have been detected with longer duration of follow‐up. In our cohort, ADT resulted in a significant reduction in both testosterone and estradiol, but it is not possible to separate the effects of these sex hormones on bone or muscle. Elegant physiologic studies selectively replacing testosterone and/or estradiol in ADT‐treated men who also received aromatase inhibitors have found that estrogen is the dominant sex steroid regulating bone resorption, whereas testosterone and estrogen are both important regulators of bone formation.^(^ ^24^, ^29^ ^)^ On the other hand, in younger men, androgen deficiency accounts for ADT‐related decreases in lean mass, muscle size, and strength, whereas estrogen deficiency primarily accounted for increase in body fat.^(^ ^30^ ^)^

The relationship between ADT, circulating sclerostin, and muscle mass has not been previously well‐characterized. In a cross‐sectional study of 240 healthy nondiabetic subjects, lean tissue mass (by dual‐energy X‐ray absorptiometry [DXA] scan) was negatively correlated with serum sclerostin (*r* = −0.245, *p* < 0.001), after adjusting for age, sex, and BMI.^(^ ^31^ ^)^ In a separate cross‐sectional study of 59 men with PCa, circulating sclerostin levels were significantly higher in those receiving ADT.^(^ ^32^ ^)^ Furthermore, serum sclerostin was inversely correlated with serum testosterone in these patients, raising the possibility that androgens may directly regulate sclerostin production.^(^ ^32^ ^)^ In a prospective cohort study of 17 male sex offenders who received ADT in the form of the androgen receptor blocker cyproterone acetate, calcium release from the skeleton due to bone resorption occurred early following sex steroid deprivation and was associated by a significant suppression in serum PTH and increase in serum sclerostin.^(^ ^33^ ^)^ In our prospective longitudinal study, we noted a similar increase in serum CTX and suppression in serum PTH. We also found that serum sclerostin increased following ADT initiation, particularly at the 6‐week time point (Fig. 1*D*). However, muscle volume (by imaging) and strength (by physical testing) did not change significantly in our study. Thus, it is difficult to directly link changes in serum sclerostin and muscle parameters in our study. Furthermore, circulating sclerostin may not entirely reflect locally produced sclerostin in the bone microenvironment, and extraskeletal sclerostin production has also been hypothesized to contribute to its circulating levels.^(^ ^34^ ^)^

In a previous report, ADT for 16 weeks in young healthy men resulted in a small but significant decrease in muscle thigh area (by CT scan) and leg‐press strength (on physical testing).^(^ ^30^ ^)^ These changes in muscle size and strength could be reversed by testosterone supplementation but not by estrogen supplementation, suggesting that androgen rather than estrogen deficiency accounts for ADT‐related decreases in lean mass, muscle size, and strength.^(^ ^30^ ^)^ Similarly, in men with PCa, ADT use was associated with a decrease in the muscle cross‐sectional area, and an increase in intramuscular lipid content (indirectly assessed as muscle attenuation by CT scan).^(^ ^35^ ^)^ Our study is the first to report on longitudinal changes in pelvic and thigh muscle fat content by MRI in ADT recipients. Our study did not substantiate the previous results of decreased attenuation on CT imaging. This might be due to a number of factors. The men in our study were physically active, which may have helped to maintain muscle mass and attenuation. Differences in imaging modality used may also be important, because Dixon fat fraction imaging is more sensitive for fat fraction quantification than CT imaging. Thus, further investigation is needed in a larger sample of population to detect any meaningful changes in these patients, which may guide future specific muscle‐strengthening protocols.

Although we hypothesized that the reduction in serum testosterone would be accompanied by a reduction in maximal force and total work capability, this was not the case in our study. Overall, maximal handgrip and lower extremity isokinetic strength was not significantly different over time with the exception of two measures: a decrease in average peak force in the dominant hand, and an increase in peak torque and total work with hip flexion under the slower isokinetic speed. We feel that the latter may have occurred because of a “practice effect” (participants becoming more comfortable with producing maximal force through isokinetic testing) or possibly from a personal desire to be stronger over time because participants seemed more internally motivated for performance during the 6‐week and 24‐week tests. Our study participants reported participating in a self‐selected regular exercise regimen (≥20 minutes of moderate intensity exercise) a median of 4 days/week at baseline and maintained this high activity level throughout the study duration. We suspect this helped to prevent loss of maximal muscle strength following ADT initiation, as was noted in prior studies of PCa patients: in one study of PCa patients with bone metastases, multimodal supervised aerobic, resistance, and flexibility exercises conducted thrice weekly resulted in self‐reported improvements in physical function and objectively measured lower body muscle strength, but no difference in lean mass, fat mass, or fatigue compared with usual care.^(^ ^36^ ^)^ In a separate study of men with PCa initiating ADT, a strategy consisting of immediate exercise training (resistance/aerobic/impact exercises started at ADT initiation) resulted in a lower decline in BMD and gain in fat mass compared with a strategy of delayed exercise initiation (started 6 months after ADT initiation).^(^ ^37^ ^)^ Similar to these prior reports, our participants experienced a decline in bone mass but not in muscle strength.

Limitations of our study include the relatively small number of participants, which may have limited our power to detect significant changes in parameters with high intraindividual variability. We also conducted multiple comparisons, raising the possibility that some of the differences we detected may have been due to chance. Our inclusion criteria required participants to be able to participate in exercise testing, and our study participants all engaged in a regular physical exercise regimen. This may have impacted our findings and our results may not be generalizable to frailer and/or sedentary ADT recipients. Finally, our analyses were limited to 24 weeks following ADT initiation, and it is likely that changes in bone and muscle strength continued or became more prominent with longer follow‐up.

In summary, in physically active men, ADT for 24 weeks results in a significant increase in bone resorption and reduction in BMD, but nonsignificant changes in gait or thigh muscle strength. Our findings highlight the need for further research into the underlying mechanisms, and the importance of preventive strategies to oppose the adverse musculoskeletal effects of falls and fractures in ADT‐treated men.

## Disclosure

Avneesh Chhabra reported the following potential conflicts of interest: Consultant: ICON Medical and TREACE Medical Concepts Inc.; Book Royalties: Jaypee, Wolters; Speaker: Siemens; Medical advisor: Image biopsy Lab Inc. All other authors have no potential conflicts of interest.

### Peer Review

The peer review history for this article is available at https://publons.com/publon/10.1002/jbm4.10573.

## Supporting information

Additional supporting information may be found online in the Supporting Information section.

**Supplemental Table S1** Longitudinal Changes in Self‐Report Questionnaires
