# Supplementary material for: Androgen Deprivation Therapy Differentially Impacts Bone and Muscle in the Short Term in Physically Active Men With Prostate Cancer
Source: JBMR Plus. 2021 Nov 15;6(1):e10573. doi: 10.1002/jbm4.10573 (PMC8770993; doi:10.1002/jbm4.10573)
Supplement: Supplementary file 1 — Supplemental Table S1 Longitudinal Changes in Self‐Report Questionnaires [file JBM4-6-e10573-s001.docx]

Supplemental Table 1. Longitudinal Changes in Self-Report Questionnaires

|  | **Baseline** | **Week 6** | **Week 24** | ***P*-value** |
| --- | --- | --- | --- | --- |
| PROMIS Physical Function | 53.9 (50.5, 57.5) | 52.9 (49.5, 56.4) | 52.9 (49.5, 56.4) | 0.37 |
| PROMIS Anxiety | 48.5 (44.3, 53.0) | 45.3 (41.4, 49.5) | 46.6 (42.6, 51.0) | 0.27 |
| PROMIS Depression | 46.3 (42.6, 50.4) | 45.6 (41.9, 49.6) | 46.8 (43.0, 50.9) | 0.19 |
| PROMIS Fatigue | 44.2 (40.0, 49.0) | 45.8 (41.4, 50.7) | 46.5 (42.0, 51.5) | 0.55 |
| PROMIS Sleep Disturbance | 44.8 (39.4, 51.0) | 45.1 (39.6, 51.2) | 47.7 (42.0, 54.3) | 0.50 |
| PROMIS Social Roles and Activities | 58.2 (54.3, 62.3) | 57.3 (53.5, 61.3) | 56.2 (52.5, 60.2) | 0.64 |
| PROMIS Pain Interference | 48.0 (44.3, 51.7) | 45.4 (41.7, 49.1) | 49.6 (45.8, 53.3) | 0.12 |
| PROMIS Pain Intensity | 1.7 (0.9, 2.5) | 1.5 (0.7, 2.3) | 1.9 (1.1, 2.8) | 0.54 |
| Activities-specific Balance Confidence Scale | 95.5 (92.5, 98.6) | 94.9 (91.9, 98.0) | 94.4 (91.4, 97.5) | 0.49 |

Data shown as least square means (95% CI)
